# Predicting Positive Affect in Infancy

**DOI:** 10.1111/desc.70192

**Published:** 2026-04-25

**Authors:** Tobias Grossmann, Halle Miller, Olivia Allison

**Affiliations:** ^1^ Department of Psychology University of Virginia Charlottesville USA

## Abstract

Positive affect (PA) is integral to health and development, yet its early origins remain largely unexplored. In this pre‐registered study (*N* = 78), we investigated whether infant‐specific biological and social‐behavioral factors predict PA in early development. Seventy‐eight typically developing infants and their mothers from a longitudinal cohort contributed complete data. At 5 months, infant *CD38* rs3796863 genotype (linked to oxytocin release), salivary cortisol levels, and social engagement during mother‐infant free play were assessed. At 7 months, infant PA was measured using the Positive Emotionality/Surgency (PEm) factor of the Infant Behavior Questionnaire‐Revised (IBQ‐R, operationalizing PA as a broader PEm temperament construct). Analysis revealed that infant PA at 7 months was predicted by infant *CD38* genotype, with AA/AC genotypes associated with higher PA; lower infant cortisol levels; and higher infant social engagement. These infant‐specific factors collectively explained 27% of the variance in infant PA. Corresponding maternal biological and behavioral measures did not predict infant PA. These findings offer novel insights into the early, multifaceted, and infant‐specific biological and behavioral foundations of PA, a crucial component of human well‐being.

**Summary:**

- Infant *CD38* genotype, cortisol, and social engagement at 5 months predict positive affect at 7 months
- Infant‐specific biological and behavioral factors account for 27% of variance in positive affect
- Maternal measures of the same variables did not predict infant positive affect
- Pre‐registered analysis validates infant‐centric model of early affective development

## 1 Introduction

A growing body of research has linked positive affect (PA) with an array of beneficial physical health outcomes. Many longitudinal studies show an association between higher levels of PA and a reduced risk of mortality, especially in older adults and those over 55, across both healthy populations and those with chronic conditions like diabetes and HIV (Pressman et al. 2019). Proposed mechanisms include PA's role in promoting healthier behaviors, such as increased physical activity, better sleep, and medication adherence, which are critical for maintaining overall health. Physiologically, PA has been associated with improved immune function, reduced stress hormone levels, and enhanced cardiovascular function; for instance, individuals with higher PA have demonstrated stronger vaccination responses, lower cortisol levels, and healthier nighttime cardiovascular activity (Pressman et al. 2019). Additionally, PA may foster social, psychological, and intellectual resources, including social support, effective coping strategies, and educational achievement, which can further contribute to physical health indirectly. Although these pathways provide a framework for understanding the health benefits of PA, further research is necessary to fully elucidate the complex mechanisms connecting PA to physical health outcomes (Pressman et al. 2019). Notably, negative affect (NA) has received considerably more research attention to date; yet PA serves both protective and promotive functions from early in life and often predicts developmental outcomes that NA does not (Abe and Izard 1999; Coffey 2018; Holder and Coleman 2009). These effects are seen primarily in adult populations, and little research has investigated the origins and predictors of PA during early human development. Understanding PA's origins in infancy can provide insight into foundational mechanisms that undergird PA as a core feature of human health.

PA in infancy, characterized by frequent expressions of joy, interest, and sociability, is associated with favorable developmental trajectories, serving as an early marker of adaptive emotional regulation and social engagement. It is important to acknowledge that PA, typically defined as the frequency of positive emotions, is conceptually related to but distinct from positive emotionality/surgency (PEm), a broader temperament construct that also encompasses approach behavior, activity level, and perceptual sensitivity (Coffey et al. 2015; Coffey 2018). The current study operationalizes infant positivity using the PEm factor of the IBQ‐R, which captures this broader suite of positively valenced temperamental traits. Where we refer to “PA” throughout, we use the term in a manner consistent with its operationalization as PEm in the developmental temperament literature. Research has shown that higher levels of PA in infancy predict greater cognitive and socio‐emotional skills in childhood and beyond (Coffey 2020; Sanson et al. 2004). In a particularly striking longitudinal study, Coffey (2020) demonstrated that infant PA at 18 months directly predicted higher childhood IQ at ages 6–8 and higher educational attainment at age 29, even after controlling for family socioeconomic status and infant intelligence. This 29‐year study provides compelling evidence that PA during infancy—before formal education begins—is linked to gold standard indicators of cognitive abilities and adult academic success. Early PA has also been linked to a greater tendency for positive social interactions and effective coping mechanisms in response to stress, laying the groundwork for emotional stability and social competence as children grow (Gartstein and Rothbart 2003; Rothbart et al. 1994). By fostering a tendency toward positive social interactions with caregivers, PA during infancy may help establish foundational socio‐emotional competencies that support lifelong well‐being and mental health (Coffey 2020; Pressman et al. 2019; Sanson et al. 2004).

The current study examined the pre‐registered hypothesis (https://osf.io/2xr6q) that PA is predicted by early identifiable individual variability in biological (oxytocin and cortisol) and social‐behavioral traits of the infant. To test these hypotheses, we conducted a pre‐registered secondary analysis combining data from a longitudinal cohort previously studied for other purposes (Krol et al. 2015, Krol et al. 2018, Krol et al. 2019). This approach allowed us to examine the combined predictive value of multiple infant‐specific factors for PA—a question not addressed in the original publications. Specifically, we selected infant oxytocin availability, basal cortisol activity, and infant social engagement as predictors because they represent distinct yet complementary pathways that may support the development of PA in early development. Oxytocin is thought to enhance the salience and reward value of social signals (Carter 2014), and genetic variation in *CD38* provides an index of individual differences in endogenous oxytocin release (Tolomeo et al. 2020). This allows us to test whether higher oxytocin availability is linked to increased sensitivity to affiliative cues and greater expression of PA. Research has demonstrated that oxytocin plays a central role in parent‐infant bonding, with increased oxytocin levels during parent‐infant contact predicting more affectionate and synchronous interactions (Feldman et al. 2010; Scatliffe et al. 2019). Notably, oxytocin administration to fathers not only increases paternal oxytocin and positive parenting behaviors, but also produces parallel increases in infant oxytocin, respiratory sinus arrhythmia, and social engagement, demonstrating the bidirectional nature of oxytocin in parent‐infant dyads (Weisman et al. 2012). In contrast, basal cortisol reflects the infant's stress physiology. Lower tonic cortisol is generally associated with greater behavioral flexibility and reduced allostatic load, suggesting that a low‐stress profile may support the expression of positive emotions (Pressman et al. 2019). Accumulating evidence indicates that prenatal and postnatal cortisol exposure shapes infant temperament, with higher maternal cortisol during pregnancy associated with increased negative reactivity and altered hypothalamic‐pituitary‐adrenal (HPA) axis regulation in infants (Davis et al. 2011; Luecken et al. 2015). Finally, infant social engagement during free play captures the behavioral expression of positive socio‐affective traits in the context of real‐time social interaction. Proactively socially engaged infants are considered to be more likely to elicit contingent caregiver responses, reinforcing positive emotion and shaping early socioemotional trajectories (Martin and Messinger 2018; Mazzocconi and Ginzburg 2022). Specifically, we hypothesized that infants who show: (1) genetic variation (*CD38*) linked to enhanced oxytocin release (AA and AC genotypes) (Krol et al. 2015), (2) reduced stress (cortisol) levels (Krol et al. 2018) and (3) increased social engagement during free play with caregiver (Krol et al. 2019), measured at 5 months of age, show greater levels of PA, operationalized as the PEm factor measured using the Infant Behavior Questionnaire (IBQ‐R) at 7 months of age (Gartstein and Rothbart 2003). Moreover, we hypothesized that infant, but not maternal measures of the same variables, will be predictive of infant PA. By integrating these predictors, we aim to test a developmental model in which higher oxytocin availability, lower cortisol, and greater social engagement act together to support the emergence of a suite of positive emotionality traits during infancy.

## 2 Method

### 2.1 Participants

This study presents a pre‐registered secondary analysis (https://osf.io/2xr6q) of existing data previously collected and reported in Krol et al. 2015, Krol et al. 2018, Krol et al. 2019). The original studies were approved by the University's Ethics Committee, and all procedures were performed in accordance with the Declaration of Helsinki. Parents provided informed consent prior to participation. Here we report a novel analysis examining the combined predictive value of infant *CD38* genotype, cortisol levels, and social engagement for PA—an analysis not conducted in the original publications. Detailed methodological procedures are available in the original publications.

The total sample consisted of 101 mother‐infant dyads who participated in the original longitudinal studies (Krol et al. 2015, Krol et al. 2018, Krol et al. 2019). Following our pre‐registered analysis plan (https://osf.io/2xr6q), only participants with complete data for all measures required for a given analysis were included. For the primary regression analysis predicting infant PA, infants were required to have complete data for all three infant predictor variables (*CD38* genotype, cortisol level, and social engagement score) as well as the outcome measure (IBQ‐R PEm), resulting in *N* = 78 (*n* = 39 female). For the pre‐registered control analysis examining maternal predictors, mothers were required to have complete data for all three maternal predictor variables (*CD38* genotype, cortisol level, and social engagement score) as well as infant PA data, resulting in *N* = 82 mother‐infant dyads.

All infants were typically developing, had a normal birth weight (>2500 g), were born full‐term (37–41 weeks), and were identified by parents as of European descent. There was no known history of autism spectrum disorder in any of the participating mothers or any of the older siblings. All mothers were still on maternity leave at the time of testing. Infants received a toy for participating, and parents were reimbursed for travel.

### 2.2 Genotyping

Infant and maternal *CD38* rs3796863 genotype was determined from saliva samples as described in detail in Krol et al. (2015). Saliva samples were collected from infants using assisted‐collection sponges and kits (CS‐2 sponges and OG‐250 kits) and from mothers using passive drool collection tubes (OG‐500 kit) from DNA Genotek, Ottawa, Canada. Samples were stored at room temperature until DNA isolation. DNA was isolated using the manual purification protocol from DNA Genotek. Genotyping of *CD38* rs3796863 was performed with a 5'‐nuclease assay using primers and probes from Applied Biosystems (TaqMan SNP Genotyping Assay). PCR was conducted with HotStarTaq Plus DNA polymerase and Q‐solution (Qiagen, Venlo, Netherlands) in a Biorad C1000 thermocycler with a CFX96 fluorescence reading module, using the following thermal protocol: Denaturation at 95°C for 5 min; followed by cycling: 95°C for 15 s, 60°C for 1 min; for 45 cycles. Following previous research (Krol et al. 2015, Krol et al. 2018, Krol et al. 2021), genotypes were grouped as AA/AC (associated with enhanced oxytocin release) versus CC (associated with reduced oxytocin release and elevated autism risk).

### 2.3 Salivary Cortisol

Infant and maternal average cortisol levels (in nmol/l) were measured from saliva as described in detail in Krol et al. (2018). Saliva samples were collected from infants and their mothers using commercially available devices (the Salimetrics Infant's Swab [Salimetrics, Suffolk, UK] and the Salivette [Sarstedt, Nümbrecht, Germany], respectively) at 5 months. Samples were stored in a −80°C freezer after each experimental session and were later transported at room temperature for analysis. Cortisol levels were analyzed at the Technical University of Dresden, Germany, using luminescence immunoassay kits purchased from IBL International (Hamburg, Germany). The functional sensitivity of the assay was 0.011 (micro)g/dL. Inter‐ and intra‐assay coefficients of variation were < 8%. For the current analysis, average basal cortisol levels at 5 months were used as predictors.

### 2.4 Infant Social Engagement

Infant and maternal social engagement was coded from a 5‐min mother‐infant free play session at 5 months as described in detail in Krol et al. (2019) and further detailed in Krol and Grossmann (2021). Infant behavior was coded to obtain an overall social engagement score based on coding infants' attention to the mother during free play, infants' mood, smiling/laughing, proximity and touch. Infant and maternal social engagement scores were z‐transformed, because they were not normally distributed (see Krol et al. 2019).

### 2.5 PA

PA at 7 months was operationalized as the PEm factor of the Infant Behavior Questionnaire‐Revised (IBQ‐R; Gartstein and Rothbart 2003). The IBQ‐R is a 191‐item parent‐report measure of infant temperament for infants between 3 and 12 months of age. Items are scored on a 7‐point Likert scale ranging from ‘never’ to ‘always’ and are averaged to calculate each subscale score. A factor analysis (Gartstein and Rothbart 2003) revealed a three‐factor structure (PEm; NA; Effortful Control) for these 14 subscales. The PEm factor used here includes the subscales: Approach, Vocal Reactivity, High‐Intensity Pleasure, Smiling and Laughter, Activity Level, and Perceptual Sensitivity. This factor captures PA frequency (via Smiling and Laughter, Vocal Reactivity) alongside broader approach‐oriented and surgency‐related traits (Approach, High‐Intensity Pleasure, Activity Level, Perceptual Sensitivity), and thus represents a broader operationalization than narrow measures of PA frequency alone (Coffey et al. 2015). This is acknowledged as a limitation of the current study (see Discussion). Mothers completed the IBQ‐R at the 7‐month assessment.

### 2.6 Maternal Variables

In addition we obtained the same measures from the infants' mothers: (1) maternal *CD38* genotype measured from saliva (Krol et al. 2015); (2) average maternal cortisol levels measured from saliva (Krol et al. 2018), (3) maternal social engagement coded during free play with primary caregiver (Krol et al. 2019). These measures were used in our pre‐registered control analysis (https://osf.io/2xr6q).

## 3 Results

**FIGURE 1:**
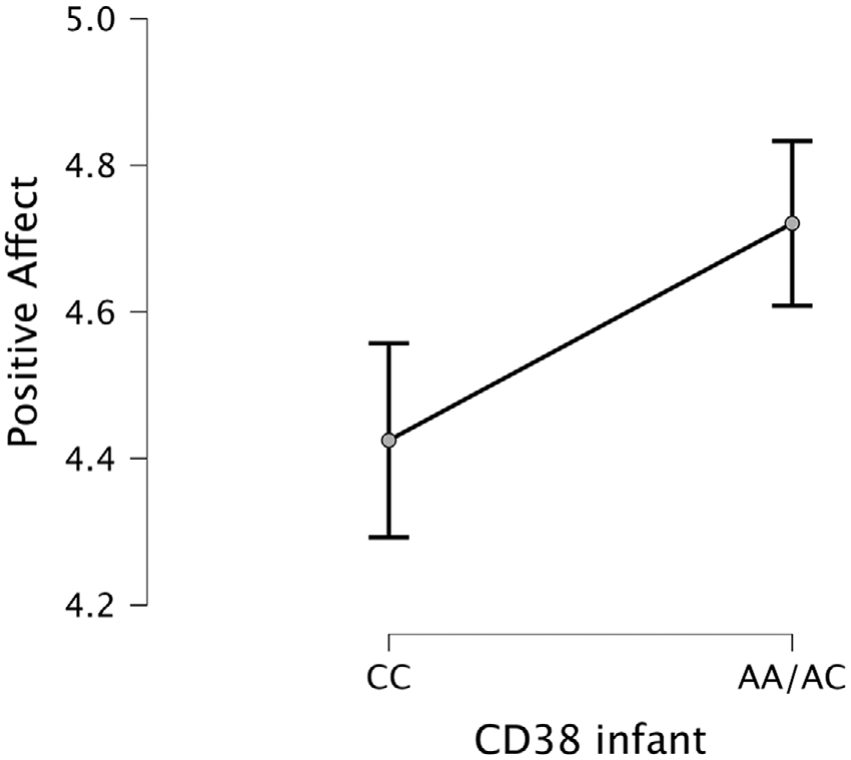
Marginal effects plot based on the multiple regression analysis showing how positive affect varies as a function of infant CD38 genotype (CC vs. AA/AC). Positive affect scores for the two genotype groups are displayed as marginal means (95% CI). *N* = 78.

**FIGURE 2:**
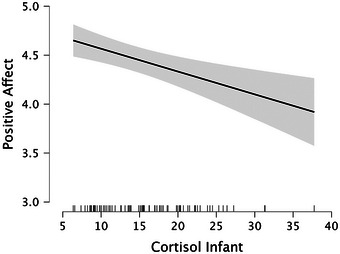
Marginal effects plot based on the multiple regression analysis showing how positive affect (95% CI) relates to infant cortisol (in nmol/l). *N* = 78.

**FIGURE 3:**
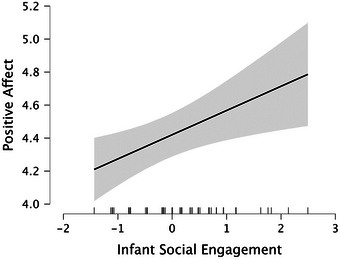
Marginal effects plot based on the multiple regression analysis showing how positive affect (95% CI) relates to infant social engagement measured during free play (z‐score). *N* = 78.

Descriptive statistics for all continuous study variables are presented in Table 1. Following our pre‐registered analysis plan (https://osf.io/2xr6q), we tested infant *CD38* genotype, salivary cortisol levels, and social engagement as predictors of infant PA in a multiple regression analysis including all 78 infants with complete data for these variables. This analysis revealed a significant effect for the multiple linear regression model, *F*(3, 74) = 9.156, *p* < 0.001, *R^2^* = 0.271. Specifically, the regression showed that infant engagement at 5 months (*ß* = 0.275, *t* = 2.712, 95% CI [0.039, 0.255], *p* = 0.008, *sr^2^* = 0.090), infant cortisol levels at 5 months (*ß* = −0.359,*t* = −3.452, 95% CI [−0.037, −0.010], *p* < 0.001, *sr^2^* = 0.139) and infant *CD38* genotype (*ß* = 0.296, *t* = 3.300, 95% CI [0.117, 0.475], *p* = 0.001, *sr^2^* = 0.128) predicted infant PA at 7 months. Confirming our predictions, infants with presumed higher levels of oxytocin (see Figure 1), lower levels of cortisol (see Figure 2) and elevated levels of social engagement (see Figure 3) at 5 months displayed higher levels of positive emotionality at 7 months, whereas infants with presumed lower levels of oxytocin (see Figure 1), higher levels of cortisol (see Figure 2) and reduced levels of social engagement (see Figure 3) at 5 months displayed lower levels of positive emotionality at 7 months. It should be noted that this design does not include a measure of PA at 5 months; thus, these results reflect prediction of positive emotionality levels at 7 months rather than evidence of change over time.

**TABLE 1:** Descriptive statistics for continuous study variables.

Variable	*N*	Mean	SD	SE
*Infant variables*				
Positive Affect (IBQ‐R PEm/Surgency)	78	4.60	0.42	0.05
Cortisol (nmol/l)	78	16.07	6.51	0.74
Social Engagement (z‐score)	78	0.03	0.79	0.09
***Maternal variables***				
Cortisol (nmol/l)	78	10.35	4.61	0.52
Social Engagement (z‐score)	78	−0.01	0.88	0.10

*Note*: Positive Affect is the outcome variable measured at 7 months; all other variables are predictors measured at 5 months. Social Engagement scores are z‐transformed. Cortisol values are in nmol/l. CD38 genotype (categorical: AA/AC vs. CC) is not shown. N reflects cases with complete data for the primary infant regression; for the maternal regression, *N* = 82.

As a pre‐registered control (https://osf.io/2xr6q), we conducted a multiple regression analysis using maternal *CD38* genotype, cortisol and social engagement as predictors of infant PA in all 82 mother‐infant dyads with complete maternal predictor data. This analysis failed to obtain any significant effects for the multiple linear regression model, *F*(3, 78) = 0.807, *p* = 0.495, *R^2^* = 0.030.

## 4 Discussion

The current study examined the pre‐registered hypothesis that PA is predicted by early identifiable individual variability in biological (oxytocin and cortisol) and social‐behavioral traits of the infant. Our results demonstrate that infants who show: (1) genetic variation (*CD38*) linked to enhanced oxytocin release (AA and AC genotypes), (2) reduced stress (cortisol) levels and (3) increased social engagement during free play with their caregiver, measured at 5 months of age, displayed greater levels of PA at 7 months of age. Together, these infant‐centric variables accounted for a substantial portion of the variance (*R^2^* = 0.271) in this key temperament trait. This predictive effect of PA was seen only for infant measures, but not for maternal measures of the same variables. In line with our hypotheses, this suggests that individual variability in biological (oxytocin and cortisol) and social‐behavioral traits of the infant, but not the mother, predicts PA in early human development.

### 4.1 CD38 Genotype and Positive Emotionality

The association between infant *CD38* genotypes (AA or AC), which are linked to more efficient oxytocin release, and higher levels of PA provides evidence for the involvement of the oxytocin system in early socio‐emotional development. This finding is consistent with the established role of the *CD38* ectoenzyme in facilitating the central release of oxytocin, a neuropeptide critical for social bonding and the modulation of social cognition (Carter 2014; Krol et al. 2015; Krol et al. 2021; Munesue et al. 2010; Tolomeo et al. 2020). The observation that the CC genotype, which is associated with reduced oxytocin release and an elevated risk for autism, corresponded with lower PA, further strengthens this interpretation (Higashida et al. 2011; Lerer et al. 2010; Munesue et al. 2010; Sung et al. 2022). This suggests an early genetic contribution to individual differences in PA, which may be mediated by the influence of the oxytocin system on the processing of socially salient and affiliative stimuli (Tolomeo et al. 2020). Our findings complement broader behavioral genetics research demonstrating that temperament dimensions, including positive emotionality and surgency, show moderate to substantial genetic influences, with heritability estimates typically ranging from 20% to 60% (Saudino 2005). Twin and adoption studies have consistently shown genetic contributions to PA and extraversion across infancy and childhood (Goldsmith et al. 1997; Planalp and Goldsmith 2020), providing a foundation for understanding how specific genetic variants like *CD38* rs3796863 may contribute to individual differences in PA.

### 4.2 Cortisol and Positive Emotionality

Furthermore, lower salivary cortisol levels in infants at 5 months predicted higher PA at 7 months, highlighting the importance of the infant's physiological stress regulation. As a primary product of the HPA axis, cortisol is a key biomarker of physiological stress (Krol et al. 2018; Pressman et al. 2019). Lower basal cortisol levels may reflect a less stressed physiological state, which is more conducive to the development of PA (Pressman et al. 2019). This finding aligns with accumulating evidence that both prenatal and postnatal cortisol exposure shapes infant temperament and emotional development. Prenatal maternal cortisol has been linked to increased negative reactivity and altered HPA axis regulation in infants (Davis et al. 2011), while postnatal infant cortisol is associated with temperamental characteristics such as negative emotionality and behavioral inhibition (Luecken et al. 2015). Research has shown that the interplay between cortisol and temperament begins early, with infant temperamental negativity interacting with prenatal maternal stress to predict infant cortisol regulation (Luecken et al. 2015). While supportive caregiving is known to buffer stress, and some research has linked lower infant cortisol to higher quality maternal care (Gunnar and Hostinar 2015), our findings indicate that it is the infant's own cortisol level, rather than the mother's cortisol level, that is predictive of PA. This suggests that lower basal cortisol may reflect effective endogenous regulation or a consistently buffered early environment, both of which would actively support the emergence of PA by freeing up internal resources. Importantly, recent work has highlighted that neurohormones and temperament interact dynamically during infant development, with cortisol and other neuroendocrine factors playing interconnected roles in shaping behavioral and emotional outcomes (Jones and Sloan 2018).

### 4.3 Social Engagement and Positive Emotionality

Consistent with the importance of early interactive experiences, higher levels of infant social engagement with their caregiver at 5 months predicted greater PA at 7 months. This finding aligns with a large body of literature emphasizing the impact of early caregiver‐infant interactions on a wide range of neurodevelopmental outcomes (Feldman 2012, 2017). Active social engagement on the part of the infant is likely to elicit more positive and contingent responses from the caregiver (Martin and Messinger 2018; Mazzocconi and Ginzburg 2022). Our findings underscore the infant's role as an active participant whose own behavioral contributions are critical in contributing to their affective development.

### 4.4 Infant‐Specificity of the Predictors

A central finding of this study is the infant‐specificity of the predictors of PA. The fact that maternal *CD38* genotype, cortisol levels, and social engagement did not predict infant PA suggests that, at this early stage of development (5–7 months), the infant's own genetic, physiological and behavioral systems are more potent contributors to their positive emotionality than the maternal characteristics assessed in this study. This challenges models of early development that overemphasize direct maternal trait‐to‐infant outcome pathways. These findings are consistent with contemporary gene‐environment interplay research demonstrating the complexity of developmental processes. Recent behavioral genetics work has shown that biological parents pass on both genotypes and home environments that correlate with their genotypes (passive gene‐environment correlation), and that family environments may suppress or facilitate the heritability of children's temperament (gene‐environment interaction) (Lemery‐Chalfant et al. 2013). The infant‐specificity of our findings suggests that at this early developmental period, infant‐driven genetic and physiological factors may predominate over simple environmental transmission from parents. This is further supported by evidence that infants’ heritable traits can evoke specific parenting behaviors, creating bidirectional effects where infant characteristics shape the caregiving environment, which in turn influences infant development (Hajal et al. 2015; Micalizzi et al. 2017). Our pattern of results—where infant but not maternal factors predict infant PA—may reflect the operation of such infant‐driven gene‐environment processes during the first year of life.

### 4.5 Strengths, Limitations, and Future Directions

The strengths of this study include its multi‐modal approach, which integrates genetic, hormonal, and behavioral data to provide a more holistic perspective on early PA development. As a pre‐registered secondary analysis of existing data, this study offers several advantages. The rigorous pre‐registration of hypotheses and analytical approach prior to data analysis reduces researcher degrees of freedom and publication bias. Additionally, the original data collection employed well‐validated methods with high‐quality measures, and our approach maximizes the scientific value of these carefully collected data while reducing participant burden by not requiring new data collection. However, the study is also subject to certain limitations. As a secondary analysis, measures were not designed specifically to test the current hypotheses. For example, we used basal cortisol levels as these were available in the original dataset, but cortisol reactivity measures would provide additional insight into stress regulation. Similarly, the timing of assessments (5 and 7 months) was determined by the original study design. Sample size was determined by available data with complete measures rather than by a priori power analysis for the current hypotheses, though the obtained sample size (*N* = 78) was sufficient to detect the observed effects. The sample was relatively homogeneous (European descent, university‐recruited families with mothers on maternity leave), and therefore, further research with more diverse samples is needed to establish the generalizability of our findings. The link between *CD38* and oxytocin was inferred based on previous research; future studies would benefit from the direct measurement of oxytocin. The use of the IBQ‐R, a parent‐report measure, is a potential source of bias; while parent‐report measures of temperament have shown convergence with more objective observational assessments (Clifford et al. 2015; Planalp and Goldsmith 2019), future research should incorporate direct observational measures of PA. It is also important to acknowledge that the biological systems we examined—oxytocin and cortisol—do not operate in isolation but interact dynamically with other neurobiological factors to shape temperament development (Jones and Sloan 2018). Additionally, longitudinal follow‐up beyond 7 months is needed to assess the long‐term outcomes of these early individual differences and to determine whether the infant PA predicted by our model continues to show the cascading benefits observed in other research, such as the association between early PA and later cognitive abilities and educational success (Coffey 2020). A critical limitation warranting explicit acknowledgment is that positive emotionality was measured only at 7 months, with no corresponding measure at 5 months or earlier. Because infants experience and express PA from birth, it is possible—and indeed likely—that individual differences in positive emotionality were already present at 5 months. Importantly, the directionality of the associations cannot be established from these data alone: for example, PA can attenuate cortisol reactivity, such that infants with higher positive emotionality at earlier ages may have had lower cortisol levels at 5 months rather than the reverse (Abe and Izard 1999; Coffey 2018). Future studies should include measures of positive emotionality at multiple time points to better characterize directional and bidirectional influences. Additionally, the use of the IBQ‐R PEm factor as the outcome measure reflects a broader construct than narrowly defined PA frequency; future work examining PA‐frequency‐specific subscales (e.g., Smiling and Laughter, Vocal Reactivity) would help clarify which components of positive emotionality are most strongly predicted by the biological and behavioral factors examined here (Coffey et al. 2015).

In conclusion, this study provides evidence for early, infant‐specific contributions to the development of PA. Infant genetic variation (*CD38*), physiological stress regulation (cortisol), and social engagement at 5 months of age are all significant predictors of PA at 7 months. These findings advance our understanding of the foundational building blocks of this vital affective trait. These results suggest that PA emerges from an integrative biological‐behavioral system already operational in the first months of life, with potential implications for early identification of individual differences in temperament and socio‐emotional development. Understanding these early determinants of PA may inform future interventions aimed at promoting optimal developmental trajectories and lifelong well‐being.

## Funding

The authors have nothing to report.

## Ethics and Consent Statement

This study is a secondary, pre‐registered analysis of previously published data (Krol et al. 2015; Krol et al. 2018; Krol et al. 2019). Parental informed consent was obtained as part of the original studies, which received ethical approval from the relevant institutional review boards. No additional consent was required for this secondary analysis.

## Conflicts of Interest

There were no conflicts of interest.

## Data Availability

The data that support the findings of this study are available from the corresponding author upon reasonable request.
